# Gender Disparities in Work and Parental Status Among Early Career Physicians

**DOI:** 10.1001/jamanetworkopen.2019.8340

**Published:** 2019-08-02

**Authors:** Elena Frank, Zhuo Zhao, Srijan Sen, Constance Guille

**Affiliations:** 1 Molecular and Behavioral Neuroscience Institute, University of Michigan, Ann Arbor; 2 Department of Psychiatry, Medical School, University of Michigan, Ann Arbor; 3 Department of Psychiatry and Behavioral Sciences, Medical University of South Carolina, Charleston

## Abstract

This survey study examines how gender disparities are associated with attrition from the workforce and how family considerations are associated with decisions about how much to work.

## Introduction

Women now make up half of incoming US medical students.^1^ While much progress has been made toward equalizing levels of entry into the field of medicine for men and women, large gaps in salary and leadership positions remain.^2,3^ To gain insight into the timing and drivers of gender gaps for the newest generation of physicians, we followed a cohort of physicians emerging from training for attrition from the workforce and the role of family considerations in decisions about how much to work.

## Methods

Physicians from multiple specialties (Table 1) who enrolled in the prospective longitudinal Intern Health Study from March 2007 to June 2013 completed an online survey about their current employment status (ie, full-time, part-time, or not employed) and gender in August 2016.^4^ All participants provided informed consent and were compensated $25. Participants working full-time were asked whether they ever considered working part-time (ie, yes or no). Except for those who reported working full-time and not having ever considered part-time work, all participants responded to an open-text question, “what specific factors influenced your decision to work full-time, part-time, or not at all?” Data were analyzed from June 2018 to June 2019. We conducted χ^2^ analyses to compare gender differences in employment status using SAS version 9.4 statistical software (SAS Institute). *P *values less than .05 were considered significant, and all tests were 2-tailed. NVivo11 software (QSR International) was used for word frequency analysis of free-text responses. Related words were thematically grouped by all of us independently, and group consensus was achieved. We followed the Strengthening the Reporting of Observational Studies in Epidemiology (STROBE) reporting guideline, and the University of Michigan institutional review board approved the study.

**Table 1.** Medical Specialties of 344 Participants

Specialty	Participants, No. (%)
Anesthesiology	15 (4.4)
Dermatology	11 (3.2)
Emergency medicine	14 (4.1)
Family medicine	13 (3.8)
Internal medicine	91 (26.5)
Internal medicine, pediatrics	12 (3.5)
Medical genetics	1 (0.3)
Missing	2 (0.6)
Neurology	6 (1.7)
Nuclear medicine	2 (0.6)
Obstetrics and gynecology	14 (4.1)
Ophthalmology	12 (3.5)
Other	37 (10.8)
Otolaryngology	5 (1.5)
Pathology, anatomic and clinical	4 (1.2)
Pediatrics	40 (11.6)
Preventive medicine	1 (0.3)
Psychiatry	25 (7.3)
Radiation oncology	2 (0.6)
Radiology, diagnostic	10 (2.9)
Surgery	22 (6.4)
Urology	5 (1.5)

## Results

Overall, 344 of 486 participants (70.8%) agreed to take part in the survey (177 [51.5%] women; median [interquartile range] age, 35.0 [34.0-36.5] years). Participants had completed their medical training a mean (SD) of 3.2 (1.7) years before completing the survey. A total 298 participants (86.6%) reported currently working full-time, 39 (11.3%) part-time, and 7 (2.0%) not at all. Women physicians were significantly more likely to report not working full-time than men physicians (40 of 177 [22.6%] vs 6 of 167 [3.6%]; odds ratio [OR], 7.83; 95% CI, 3.22-19.04) (Table 2), and differences were even greater among women with children compared with men with children (33 of 108 [30.6%] vs 5 of 109 [4.59%]; OR, 9.15; 95% CI, 3.41-24.54). A 9.6% gender gap in full-time employment (24 of 27 men [88.9%] vs 23 of 29 women [79.3%]) was present in the first year after training and grew to 38.7% by 6 years after training (21 of 21 men [100%] vs 19 of 31 women [61.3%]). Of physicians currently working full-time, women were significantly more likely to report considering part-time work compared with men (87 of 135 [64.4%] vs 33 of 156 [21.2%]; OR, 6.76; 95% CI, 4.01-11.38) (Table 2) and differences were even greater among women with children compared with men with children (52 of 74 [70.3%] vs 19 of 100 [19.0%]; OR, 10.08; 95% CI, 4.98-20.41). Women were more likely than men to mention family as a factor influencing their work status considerations (33 of 87 [37.9%] vs 5 of 33 [15.2%]; OR, 3.42; 95% CI, 1.20-9.74). Overall, 31 of 40 women physicians (77.5%) currently working part-time or not at all cited family as the factor that influenced their employment status decision.

**Table 2.** Current Work Hours by Gender and Years Since Training

Years Since Completing Residency Training	No./Total No. (%)		Odds Ratio (95% CI)
	Male (n = 167)	Female (n = 177)	
**Currently Working Full-time**			
1	24/27 (88.9)	23/29 (79.3)	2.09 (0.47-9.35)
2	45/47 (95.7)	35/40 (87.5)	3.21 (0.59-17.57)
3	36/36 (100)	23/26 (88.5)	10.87 (4.20-28.15)
4	23/23 (100)	21/29 (72.4)	18.85 (7.40-46.66)
5	12/12 (100)	16/22 (72.7)	9.85 (3.85-25.18)
6	21/21 (100)	19/31 (61.3)	27.56 (11.04-68.60)
**Currently Working Full-time but Considering Part-time**			
1	5/24 (21.7)	14/22 (63.6)	6.65 (1.79-24.73)
2	10/44 (22.7)	22/35 (62.9)	5.75 (2.15-15.38)
3	7/34 (20.6)	14/23 (60.9)	6.00 (1.84-19.53)
4	4/22 (18.2)	15/20 (75.0)	13.50 (3.07-59.46)
5	3/12 (25.0)	9/16 (56.3)	3.86 (0.75-19.84)
6	4/20 (20.0)	13/19 (68.4)	8.67 (2.01-37.38)

## Discussion

The current generation of young women physicians is the first to come of age in an era when they are not a clear minority in medicine, when women have workplace rights, and when attitudes toward gender roles are increasingly egalitarian. Yet, when it comes to balancing a medical career and a family, our findings suggest that not much has changed. Today’s young women physicians still struggle to have it all and therefore reduce their work hours at substantially higher rates than men in an effort to reduce work-family conflict.^3,4,5,6^ While our qualitative data support the quantitative responses regarding work-family conflict, future studies using a validated assessment tool, as opposed to a text-based response, may yield additional information.

More notable is a substantial gender disparity in work status that emerges immediately following medical training. Within 6 years, almost three-quarters of women physicians reported reducing work hours to part-time or considering part-time work. The emergence of this gap so early in physicians’ careers may contribute to later gender inequities in compensation and promotion and suggests the importance of expanding social and institutional support for work-family balance moving forward. Until policies and a culture allowing women and men to be both parents and physicians are created, women are less likely to be retained and to advance.
